# Exonic and Intronic *WNT10A* Variants Isolated from Korean Children with Non-Syndromic Tooth Agenesis

**DOI:** 10.3390/diagnostics15030310

**Published:** 2025-01-28

**Authors:** Yeonjin Ju, Joo Yeon Lee, Woochang Hwang, Jonghyun Shin, Hyung-Sik Kim, Junho K. Hur, Eungyung Lee

**Affiliations:** 1 Department of Pediatric Dentistry, School of Dentistry, Dental and Life Science Institute, Pusan National University, Yangsan 50612, Republic of Korea; yeonjj94@pusan.ac.kr (Y.J.); jonghyuns@pusan.ac.kr (J.S.); 2 Department of Pediatric Dentistry, Dental Research Institute, Pusan National University Dental Hospital, Yangsan 50612, Republic of Korea; 3 New Drug Development Center, OSONG Medical Innovation Foundation, Cheongju 28160, Republic of Korea; hyuljy17@hanyang.ac.kr; 4 Department of Biomedical Science, Graduate School of Medicine, Hanyang University, Seoul 04763, Republic of Korea; 5 Department of Pre-Medicine, College of Medicine, Hanyang University, Seoul 04763, Republic of Korea; whwang22@hanyang.ac.kr; 6 Hanyang Institute of Bioscience and Biotechnology, Hanyang University, Seoul 04763, Republic of Korea; 7 Department of Oral Biochemistry, School of Dentistry, Pusan National University, Yangsan 50612, Republic of Korea; hskimcell@pusan.ac.kr; 8 Dental and Life Science Institute, Pusan National University, Yangsan 50612, Republic of Korea; 9 Department of Genetics, College of Medicine, Hanyang University, Seoul 04763, Republic of Korea

**Keywords:** tooth agenesis, genetic variation, polymorphism, *WNT10A*

## Abstract

**Background/Objectives**: Tooth agenesis (TA) is a developmental anomaly prevalent in humans. It is particularly significant in children and adolescents because it is related to esthetic, physiological, and functional problems, including malocclusion, periodontal damage, and insufficient alveolar growth. *WNT10A* mutations have been identified as the main genetic alterations associated with tooth agenesis. Most previous studies have investigated *WNT10A* mutations in patients with tooth agenesis using single nucleotide polymorphism (SNP) arrays or exome sequencing. In this study, we conducted a comprehensive profiling of mutations within the exons and introns of *WNT10A* in Korean patients with non-syndromic tooth agenesis. **Methods**: Saliva samples were collected from Korean children and adolescents with non-syndromic tooth agenesis. Tagmentation-based sequencing was conducted to acquire mutation information for all exonic and intronic bases of the *WNT10A* gene. **Results**: Mutations were detected exclusively in the patient samples: 629C>G and 1100C>T in exon 1, 1977T>C in intron 1, 10256C>T and 10382G>A in exon 3, and 15953G>A in intron 4. Additional mutations were also observed at high ratios in the patient samples. **Conclusions**: The mutations identified in this study differ from previous findings. These results may provide useful information for understanding the pathogenicity of *WNT10A* mutations in Korean patients with tooth agenesis and support future diagnostic and therapeutic approaches.

## 1. Introduction

Tooth agenesis (TA) is a common developmental anomaly in humans, with significant differences in the number and type of teeth involved [1]. Tooth agenesis can be classified into anodontia, hypodontia, and oligodontia. Hypodontia refers to the agenesis of five or fewer teeth, while oligodontia refers to six or more congenitally missing teeth, excluding the third molars [2,3]. Anodontia occurs when all permanent teeth are missing. Tooth agenesis may occur as a part of a recognized genetic syndrome or as an isolated non-syndromic trait. Anodontia occurs almost exclusively in syndromic cases [4,5]. 

Although tooth agenesis can be affected in both the primary and permanent dentition, it is considered rare in the deciduous dentition and is not as common as in the permanent dentition [1,6]. The prevalence of non-syndromic tooth agenesis ranges from 3% to 10% in hypodontia, whereas oligodontia shows a prevalence of 0.1–0.5% [4]. Its prevalence is higher in Asians than in Caucasians or African Americans [4,7]. Chung et al. reported that the prevalence of hypodontia in Koreans is 11.2% [8]. Tooth agenesis in children and adolescents is important because esthetic, physiological, and functional problems such as malocclusion, lack of alveolar growth, and periodontal damage can be caused by hypodontia [9]. 

Several previous studies have investigated genetic mutations associated with tooth agenesis. These included muscle segment homeobox 1 (*MSX1*, OMIM 142983), paired box 9 (*PAX9*, OMIM 167416), interferon regulatory factor 6 (*IRF6*, OMIM 607199), axis inhibition protein 2 (*AXIN2*, OMIM 604025), ectodysplasin-A (*EDA*, OMIM 300451), ectodysplasin-A receptor (*EDAR*, OMIM 604095), and ectodysplasin-A receptor-associated adapter (*EDARADD*, OMIM 606603) [10,11,12,13,14,15,16,17,18,19]. *MSX1* is a transcription factor involved in the development of the teeth, jawbones, and oral tissues. It interacts with the bone morphogenetic protein 4 (BMP4) signaling pathway to regulate tooth bud development during the tooth formation process [16,20]. *PAX9* interacts with *MSX1* to regulate tooth germ formation and root development and mediates epithelial-mesenchymal interactions. *PAX9* and *MSX1* are primarily associated with molar agenesis [17,21]. *IRF6* regulates the growth and differentiation of epithelial cells and has been implicated in certain cases of tooth agenesis associated with cleft lip and palate [10]. *AXIN2* functions as an inhibitor of the *WNT*/β-catenin pathway and has been particularly associated with agenesis of incisors and second premolars [18,22]. *EDA, EDAR,* and *EDARADD* are involved in tooth agenesis and abnormalities in tooth morphology through the nuclear factor-κB (NF-κB) signaling pathway [15,23,24]. *WNT10A* (chromosome 2q35, OMIM 606268) has been investigated in many studies, and more than 50% of the cases of tooth agenesis have been attributed to *WNT10A* mutations [25,26]. 

Most of these studies have used single nucleotide polymorphism (SNP) arrays or exome sequencing to identify gene mutations. However, in these analyses, SNP arrays could only detect known mutations in patient samples, and exome sequencing could only analyze mutations in the exons of *WNT10A*. The *WNT10A* gene consists of four exons and four introns, making it likely that mutations located in intronic regions are overlooked by these methods [27]. Therefore, it is highly likely that the mutations in *WNT10A* identified using these methods provide limited information. In addition, because the genetic characteristics of tooth agenesis can vary between races, it is inappropriate to apply the known SNP array results to Koreans. 

This study aimed to perform the mutational profiling of *WNT10A* in saliva samples from Korean children and adolescents with non-syndromic tooth agenesis. Tagmentation-based next-generation sequencing (NGS) methods were used to obtain sequence information from thousands of base pairs of *WNT10A* DNA.

## 2. Materials and Methods

### 2.1. Subject Selection and Clinical Evaluation

This study was performed in accordance with the guidelines of the Declaration of Helsinki and approved by the Institutional Review Board of Pusan National University Dental Hospital (PNUDH-2021-06-033, 27 July 2021). Informed consent was obtained from all participants. The assent of children aged 6 years or older was additionally obtained and documented. 

Sixty patients who visited the Department of Pediatric Dentistry at the Pusan National University Dental Hospital participated in this study. Patients with systemic diseases and disabilities and patients who had mesiodens or supernumerary teeth were excluded from the study. The oral examinations were performed by a pediatric dentist (E.L.). Panoramic radiographs were obtained from all the subjects to confirm the diagnosis of tooth agenesis. Tooth agenesis codes (TAC) were recorded for patients diagnosed with hypodontia or oligodontia. The teeth were numbered 1 to 8 based on the Fédération Dentaire Internationale (FDI) system to assign TAC scores. 2^(n−1)^ could be used to calculate the tooth value, where n was the tooth number. All values were added to determine the TAC score, which is related to the missing teeth in this pattern [3,28,29]. Finally, saliva samples were collected from all subjects for DNA extraction. The subject group comprised 40 participants, whereas the control group comprised 20.

#### 2.1.1. Inclusion Criteria

- Patients diagnosed with tooth agenesis in the permanent dentition
- Patients with available panoramic radiographs

#### 2.1.2. Exclusion Criteria

- Low-quality radiographic records
- Patients with systemic diseases and disabilities
- Patients with mesiodens or other supernumerary teeth

### 2.2. Genomic DNA (gDNA) Extraction

Saliva samples (up to 2 mL) were collected using an OG-600 kit (DNA Genotek, Ottawa, ON, Canada). Saliva was extracted using a QIAamp DNA Mini Kit (Qiagen, Hilden, Germany). After transferring the patient’s saliva from the OG-600 to a new Eppendorf tube, ATL buffer was added until the total volume reached 100 µL. 10 µL of Proteinase K and 100 µL of AL buffer were added, followed by vortexing. The mixture was incubated at 56 °C for 10 min and centrifuged again. Fifty microliters of ice-cold, molecular biology-grade ethanol (96–100%) were added, vortexed, and incubated for 3-min incubation at room temperature. The entire lysate was transferred to a QIAamp MinElute (Qiagen, Hilden, Germany) column (in a 2 mL collection tube) and centrifuged at 6000× *g* (8000 rpm) for 1 min. The waste collected in the collection tube below the column was discarded, and 500 µL of AW1 buffer was added, followed by another centrifugation at 6000× *g* (8000 rpm) for 1 min. The waste was discarded again, and 500 µL of AW2 buffer was added, followed by another centrifugation at 6000× *g* (8000 rpm) for 1 min. The waste is discarded. To dry the membrane, the column was centrifuged at 20,000× *g* (14,000 rpm) for 3 min. The collection tube below the column was replaced with a new Eppendorf tube, and 20 µL of nuclease-free water was added to the membrane. After incubation for 1 min at room temperature, the tubes were centrifuged at 20,000× *g* (14,000 rpm) for 1 min. The concentration of the eluted gDNA was measured using a NanoDrop^TM^ spectrophotometer (Thermo Fischer Scientific, Waltham, MA, USA).

### 2.3. Polymerase Chain Reaction (PCR)

The sequence for *WNT10A-001* (ENST00000258411, CCDS2426) was extended by adding 500 bp putative promoter regions at both ends, resulting in a total length of 20,219 bp. Extended sequences were divided into four segments. PCR was performed on each of these segments, and the primer sequences are listed in Table 1. These segments were designed with some overlap for validation purposes (the first segment was 2816 bp, the second segment was 8475 bp, the third segment was 3520 bp, and the fourth segment was 7247 bp). PCR reactions were set up with a volume of 50 µL, using 50 ng of gDNA, a 3 µL primer mix (10 pmol), and KOD One™ PCR Master Mix (TOYOBO, Osaka, Japan) as the polymerase. Only the first and third segments were subjected to 5X Band Doctor (SolGent, Daejeon, Republic of Korea) treatment. The PCR process involved denaturation at 98 °C for 2 min. In each cycle, denaturation was conducted at 98°C for 10 s, annealing at 60 °C for 5 s, and extension at 68 °C. The duration of the extension step varied for each segment (first segment: 15 s, second segment: 45 s, third segment: 20 s, fourth segment: 36 s). A total of 30 cycles were performed. The final extension was performed at 68°C for 5 min.

### 2.4. Sequencing and Mutation Analysis

Sequencing was conducted using BTSeq™ (Celemics, Seoul, Republic of Korea). Sequencing data in the FASTQ format were aligned using a program called Benchling^TM^ (Benchling, San Francisco, CA, USA). Comparing the sequences obtained from the reference genome *WNT10A-001* (ENST00000258411, CCDS2426) with sequences from the samples, any differences were considered mutations. For samples in which mutations were not explicitly labeled, if the lead (prevalence) indicated the presence of the mutation in a heterozygous form, it was categorized as a heterozygous mutation.

### 2.5. Statistical Analysis

*T*-tests were performed to determine whether there was a difference in age between the tooth agenesis and control groups.

## 3. Results

Table 2 summarizes the characteristics of the study participants. Of the 60 participants, 40 were diagnosed with oligodontia, and 20 were controls. The mean age of participants was 9.8 years. The mean age of the tooth agenesis group was 9.6 years, while that of the control group was 10.1 years. However, these differences were not statistically significant.

Table 3 and Table 4 present the distribution of TAC scores in the TA group. The number of congenitally missing teeth, excluding third molars, was determined using panoramic radiographs. The TAC scores ranged from 2 to 110, with higher scores indicating a greater number of missing teeth in the posterior area. We grouped the patient data into three TAC score groups (0–8, 8–32, and 32–110). Among girls, the group with a lower TAC score (0–8) had a higher number of patients (10 individuals). However, among boys, all three TAC score groups had the same number of patients. 

Specific mutations were found only in TA samples in exons 1 and 3 and introns 1 and 4 (Table 5). Mutations in exon 1 included c.629T>G and c.1100C>T, each of which was observed in one subject. A mutation in exon 3 results in an amino acid substitution. c.10256C>T resulted in p.Arg171Thr, and c.10382G>A resulted in p.Gly213Ser, which was observed in three subjects. Notably, one subject (TA-005) harbored both mutations in exon 3.

Some mutations were found in the majority of TA samples and in some controls (Table 6). The c.621 mutations in exon 1 were found in 22 of the 40 TA samples and 5 of the 20 controls. Among these, c.621C>del was found in 13 TA subjects and one control, and c.621C>G was found in nine subjects and four controls. The c.14596T>G mutation in intron 4 was detected in 23 subjects and 5 controls.

Certain mutations were found only in TA samples (Table 7). Among the mutations in the exon region, patients with mutations in exon 1 had two missing teeth in the anterior region, whereas patients with mutations in exon 3 mostly had missing teeth in the posterior region, indicating a tendency toward a higher number of missing teeth. TA subject TA-005, who had both mutations in exon 3, had the most missing teeth among the patients with *WNT10A* mutations and had the highest TAC score of 96. Patients with mutations in the intron region showed milder phenotypes with four or fewer missing teeth in the anterior or posterior regions.

## 4. Discussion

Genetic causes of tooth agenesis have been extensively studied. However, limited research has been conducted in Korean children and adolescents. Although the severity based on the number of missing teeth per individual was modest, the prevalence of hypodontia in the Korean orthodontic population in this study was 11.2%, which is marginally higher than that reported for Caucasian and Japanese individuals [8,30]. In particular, clinical management is required to establish long-term treatment plans for children and adolescents with non-syndromic tooth agenesis.

Tooth agenesis is a highly heritable trait that involves various genes. Among them, the *WNT*/β-catenin pathway plays a significant role in the tooth morphogenesis of both primary and secondary dentition. Within the context of tooth morphogenesis, the key mediator of *WNT* signaling is *WNT10A*, which is implicated in inherited tooth agenesis [12,19,31,32]. It has been reported that over 50% of oligodontia probands have *WNT10A* mutations [25,26]; according to a population-based cohort study, approximately 28% of oligodontia probands have *WNT10A* mutations [14]. Therefore, this study aimed to analyze the *WNT10A* mutation, which is considered the most prevalent gene in oligodontia.

Various methods, including saliva and blood analyses, can be used for genomic DNA collection. Among these, analyzing genomic DNA from saliva has the advantage of being convenient and noninvasive for pediatric patients. In a previous study focusing on gene mutations in Koreans, Park et al. analyzed *WNT10A* in the salivary genomic DNA of two families with oligodontia. They found not only a common paternal c.364A>T mutation but also maternal c.511C > T and c.637G > A [33]. Song et al. conducted Sanger sequencing in a Korean family with non-syndromic oligodontia. The results revealed no pathogenic variations in *WNT10A*. Heterozygous variants were identified in the thrombospondin-type laminin G domain and EAR repeats (*TSPEAR*, OMIM 612920) [34]. In the present study, we identified four exonic and two intronic mutations: c.629T>G and c.1100C>T in exon 1, c.10256C>T and c.10382G>A in exon 3, c.1977T>C in intron 1, and c.15953G>A in intron 4. These mutations were not identified in previous studies.

Next-generation sequencing (NGS) is a high-throughput technique. NGS sequences an organism’s genome by fragmenting it into numerous pieces, concurrently reading each piece, and then reassembling them computationally. This method rapidly decodes vast amounts of genomic information using massive parallel sequencing [35,36]. This study utilized an NGS method, BTSeq™, to identify mutations in the exons and introns of the *WNT10A* gene. Previous studies that identified *WNT10A* mutations associated with tooth agenesis only identified mutations in exonic regions, excluding introns, or performed whole-genome sequencing (WGS), which resulted in significant data size and cost limitations. In contrast, BTSeq™, applied in this study, offered targeted sequencing of up to 20 kb, including introns, by generating libraries efficiently. This method might be employed not only for oligodontia but also for the rapid and efficient analysis of gene mutations implicated in rare dental conditions, contributing to the field of dentistry.

Among the 40 subjects with TA, seven (17.5%) had exon mutations. Previous studies reported that approximately 28% of subjects have *WNT10A* mutations [14]; however, in this study, a lower frequency of 17.5% was observed. This low frequency may be partially attributed to the small number of participants. Two mutations in exon 1 were found in one subject, whereas two mutations in exon 3 were found in three subjects. One subject had both mutations in exon 3 and had the highest number of missing teeth among the subjects with *WNT10A* mutations. Mutations in exon 3 are associated with a relatively high number of missing teeth in the posterior region, potentially due to the amino acid substitutions caused by these mutations, suggesting that this region may be related to molar tooth development. In the intronic region, two mutations were detected in four patients. These mutations did not show a distinct correlation with the number or location of the missing teeth.

This study is significant not only for observing exon mutations but also for identifying intron mutations. As these splice-altering variants may affect splicing factor recognition, they may lead to alternative splicing by creating pathogenic pseudo-exons or extending existing exons [37]. Some intronic variants with splice alterations cause dystrophinopathy (OMIM 310200 and 300376), neurofibromatosis type I (OMIM 162200), and inherited retinal diseases [38]. However, research on intronic mutations and their effects on *WNT10A* remains incomplete, and further studies are required.

This study had several limitations. First, we only observed mutations in the WNT10A gene. However, it is necessary to identify different types of gene mutations depending on the location of tooth agenesis. Broader genetic analysis could provide a more comprehensive understanding of the genetic factors underlying this condition. Secondly, the sample size was small. To identify the racial characteristics associated with the correlation between *WNT10A* mutations and tooth agenesis, a larger number of subjects is needed. In addition, there may be regional differences in the prevalence of tooth agenesis. Furthermore, a potential selection bias may have been present as the study was conducted at a single institution, which may limit the generalizability of the findings to other populations. Including multiple centers and a more diverse population would enhance the external validity of the study. Finally, the study primarily assessed tooth agenesis, but incomplete clinical phenotype assessment is another limitation, as other associated dental anomalies, such as microdontia, delayed eruption, and enamel hypoplasia, were not analyzed. A more detailed evaluation of the dental phenotype could further clarify the role of *WNT10A* mutations in dental abnormalities.

Based on previous research, future studies should investigate the interactions between *WNT10A* and other genes involved in tooth agenesis. A multigene approach could provide a more comprehensive understanding of the genetic basis of non-syndromic tooth agenesis. Additionally, this study did not assess the long-term clinical outcomes. Future longitudinal cohort studies should examine how *WNT10A* mutations influence tooth development and other clinical outcomes.

## 5. Conclusions

In this study, we performed mutational profiling of *WNT10A* in the saliva of Korean children and adolescents with non-syndromic tooth agenesis. We identified four exonic mutations (c.629T>G and c.1100C>T in exon 1 and c.10256C>T and c.10382G>A in exon 3) that resulted in amino acid changes and two intronic mutations (c.1977T>C in intron 1 and c.15953G>A in intron 4), some of which have not been previously reported. Notably, mutations in exon 3 (c.10256C>T and c.10382G>A) were associated with a higher number of missing posterior teeth, suggesting a possible genotype-phenotype correlation. Longitudinal studies are required to assess how *WNT10A* mutations influence long-term dental development and clinical outcomes. Further investigation of intronic mutations and their potential effects on alternative splicing will provide deeper insights into the molecular mechanisms underlying tooth agenesis. 

## Figures and Tables

**Table 1:** *WNT10A* primer sequence.

Region	Sequence
Region 1	Forward: 5′-TCTCTCTAACGCCTCCTCCCA
	Reverse: 5′-TGACCCAGGAGTCCAGTTCT
Region 2	Forward: 5′-GGCAGGATGATTGTGAGGAG
	Reverse: 5′-CGTGGTCCTCAGAAGAGAGG
Region 3	Forward: 5′-TTCCTTGTGCCAGACTCTCC
	Reverse: 5′-CCTCTTCCCAAGAGCCAAG
Region 4	Forward: 5′-GCGTTTGCCTCTGTATAATGG
	Reverse: 5′-AGGAGGTTGAGGCAGTGCAAT

**Table 2:** Subject distributions.

	Gender (*n*)		Age (Years)	Total (*n*)	*p*
	F	M			
TA	21	19	9.6	40	0.482 *
Control	12	8	10.1	20	
Total	33	27	9.8	60	

* *p*-value was derived from the *t*-test.

**Table 3:** Missing teeth and tooth agenesis codes (TAC) in patients with tooth agenesis.

Subject	Missing Teeth (*n*)			TAC Score
	Anterior	Posterior	Total	
TA-001	2	0	2	4
TA-002	0	7	7	88
TA-003	0	1	1	16
TA-004	0	1	1	16
TA-005	0	8	8	96
TA-006	3	0	3	6
TA-007	0	1	1	16
TA-008	2	0	2	4
TA-009	1	0	1	2
TA-010	1	6	7	66
TA-011	0	8	8	96
TA-012	0	1	1	16
TA-013	3	0	3	6
TA-014	0	1	1	16
TA-015	0	1	1	16
TA-016	2	0	2	4
TA-017	2	2	4	32
TA-018	0	5	5	72
TA-019	0	2	2	24
TA-020	3	1	4	25
TA-021	2	0	2	4
TA-022	0	7	7	88
TA-023	4	5	9	76
TA-024	8	7	15	110
TA-025	2	0	2	4
TA-026	2	0	2	4
TA-027	1	8	9	98
TA-028	2	0	2	4
TA-029	2	0	2	6
TA-030	1	0	1	2
TA-031	2	0	2	2
TA-032	0	7	7	88
TA-033	2	0	2	4
TA-034	1	0	1	2
TA-035	0	2	2	32
TA-036	2	0	2	4
TA-037	0	8	8	96
TA-038	1	0	1	4
TA-039	0	0	0	16
TA-040	0	4	4	64

**Table 4:** Distribution of TAC score by gender.

Gender	0 < TAC ≤ 8	8 < TAC ≤ 32	32 < TAC ≤ 200	Total
F	7	7	7	21
M	10	5	4	19
Total	17	12	11	40

**Table 5:** Mutations detected exclusively in TA samples.

Region	Mutation	Amino Acid Change	Subject
Exon 1	c.629T>G	-	TA-029
	c.1100C>T	-	TA-021 *
Intron 1	c.1977T>C	-	TA-017 *, 019 *, 020 *
Exon 3	c.10256C>T	p.Arg171Thr	TA-002 *, 005 *, 014 *
	c.10382G>A	p.Gly213Ser	TA-005 *, 010 *, 018 *
Intron 4	c.15953G>A	-	TA-009 *

* Heterozygous mutation.

**Table 6:** Mutations detected in most TA samples and some control samples.

Region	Mutation	Amino Acid Change	Subject (Total *n* = 60)	
			TA (*n* = 40)	Control (*n* = 20)
Exon 1	c.621C>del	-	13 (32.5%)	1 (5%)
	c.621C>G	-	9 (22.5%)	4 (20%)
Intron 4	c.14596T>G	-	23 (57.5%)	5 (25%)

**Table 7:** Mutations detected exclusively in TA samples and TAC scores.

Region	Mutation	Amino Acid Change	Subject	Anterior (*n*)	Posterior (*n*)	Missing Teeth (*n*)	TAC Score
Exon 1	c.629T>G	-	TA-029	2	0	2	6
	c.1100C>T	-	TA-021	2	0	2	4
Exon 3	c.10256C>T	p.Arg171Thr	TA-002	0	7	7	88
			TA-005	0	8	8	96
			TA-014	0	1	1	16
	c.10382G>A	p.Gly213Ser	TA-005	0	8	8	96
			TA-010	1	6	7	66
			TA-018	0	5	5	72
Intron 1	c.1977T>C	-	TA-017	2	2	4	32
			TA-019	0	2	2	24
			TA-020	3	1	4	25
Intron 4	c.15953G>A	-	TA-009	1	0	1	2

## Data Availability

All data are available from the corresponding author upon request.

## Abbreviations

*AXIN2*	Axis inhibition protein 2
BMP4	Bone morphogenetic protein 4
*EDA*	Ectodysplasin-A
*EDAR*	Ectodysplasin-A receptor
*EDARADD*	Ectodysplasin-A receptor-associated adapter
FDI	Fédération Dentaire Internationale
gDNA	Genomic DNA
*IRF6*	Interferon regulatory factor 6
*MSX1*	Muscle segment homeobox 1
NF-κB	Nuclear factor-κB
NGS	Next-generation sequencing
*PAX9*	Paired box 9
PCR	Polymerase Chain Reaction
SNP	Single nucleotide polymorphism
TA	Tooth agenesis
TAC	Tooth agenesis codes
*TSPEAR*	Thrombospondin-type laminin G domain and EAR repeats
WGS	Whole-genome sequencing
